# Sex differences in muscle morphology between male and female sprinters

**DOI:** 10.1152/japplphysiol.00009.2023

**Published:** 2024-04-25

**Authors:** Robert Miller, Thomas G. Balshaw, Garry J. Massey, Sumiaki Maeo, Marcel B. Lanza, Bill Haug, Michael Johnston, Sam J. Allen, Jonathan P. Folland

**Affiliations:** ^1^School of Sport, Exercise and Health Sciences, Loughborough University, Loughborough, United Kingdom; ^2^British Athletics, Loughborough University, Loughborough, United Kingdom; ^3^School of Sport and Health Sciences, University of Exeter, Devon, United Kingdom; ^4^Faculty of Sport and Health Science, Ritsumeikan University, Kusatsu, Japan; ^5^Department of Physical Therapy and Rehabilitation Science, University of Maryland Baltimore, Baltimore, Maryland, United States; ^6^Applied Sports Technology Exercise and Medicine Research Centre, Swansea University, Swansea, United Kingdom; ^7^National Institute of Health and Care Research (NIHR) Leicester Biomedical Research Centre, Loughborough University, Loughborough, United Kingdom

**Keywords:** muscle morphology, sex differences, sprinters

## Abstract

There is a marked difference between males and females in sprint running performance, yet a comprehensive investigation of sex differences in the muscle morphology of sprinters, which could explain the performance differences, remains to be completed. This study compared muscle volumes of 23 individual leg muscles and 5 functional muscle groups, assessed with 3 T magnetic resonance imaging, between male (*n* = 31) and female (*n* = 22) sprinters, as well as subgroups of elite males (EM, *n* = 5), elite females (EF, *n* = 5), and performance-matched (to elite females) males (PMM_EF_, *n* = 6). Differences in muscle volume distribution between EM, EF, and unathletic male (UM) controls were also assessed. For the full cohorts, male sprinters were more muscular than their female counterparts, but the differences were nonuniform and anatomically variable, with the largest differences in the hip extensors and flexors. However, among elite sprinters the sex differences in the volume of the functional muscle groups were almost uniform (absolute volume +47–53%), and the muscle volume distribution of EM was more similar to EF than to UM (*P* < 0.039). For PMM_EF_, relative hip extensor volume, but not stature or percent body fat, differentiated for performance (PMM_EF_ and EF < EM) rather than sex. In conclusion, although the full cohorts of sprinters showed a marked sex difference in the amount and distribution of muscle mass, elite sprinters appeared to be selected for a common muscle distribution phenotype that for these elite subgroups was a stronger effect than that of sex. Relative hip extensor muscle volume, rather than stature, percent body fat, or total relative muscle volume, appeared to be the primary determinant of the sex difference in performance.

**NEW & NOTEWORTHY** We present novel evidence suggesting muscle volume, specifically relative hip extensor volume, may be a primary deterministic variable for the sex difference in sprint performance, such that with matched sprint times, male and female sprinters may be expected to have equivalent muscle morphology. We highlight striking similarities in distribution of leg muscle mass between elite male and female sprinters and provide evidence for the existence of a muscular distribution phenotype specific to elite sprinters, irrespective of sex.

## Graphical abstract

**Figure:**
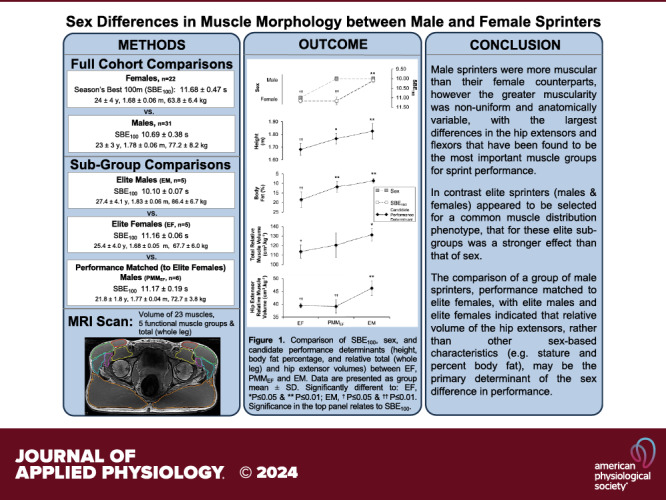

## INTRODUCTION

The 100-m sprint is one of the most prestigious events in track and field athletics, epitomizing the speed capabilities of human beings in bipedal locomotion. It is a common observation that there is a marked difference between males and females in sprint running performance as exemplified by the 100-m sprint world record being 9% faster for males (9.58 s) than for females (10.49 s). Several characteristics have been suggested to contribute to the performance difference between males and females, such as anatomical [e.g., height, limb lengths, and body composition (1)]; physiological [e.g., hormonal and enzymatic factors (2)]; and biomechanical [e.g., stride length and frequency (3)] factors. Success in sprint running is highly dependent on the generation of large forces relative to body mass (4) at high muscle fiber shortening velocities (5); thus, it is accepted that muscular power (the combination of force and velocity) of the lower body plays a considerable role in sprint running success (6, 7). Sexual dimorphism in humans is such that males can produce greater neuromuscular power, both in absolute terms and relative to body mass, than females (8, 9), in large part due to substantial differences in absolute muscle volume (9, 10). Therefore, muscle volume could explain the sexual dimorphism in sprint performance. However, to date the differences in the muscle volume between male and female sprinters have received little attention, and whether any differences in muscle volume may explain performance differences has not been investigated.

Previous research, including recent work from our laboratory (11, 12), has utilized magnetic resonance imaging (MRI), considered the “gold-standard” method of in vivo muscle volume measurement (13), and has investigated how the muscularity of either male (12, 14–18) or female (11) sprint athletes was related to sprint performance. However, to date the specific differences in muscle volumes between male and female sprinters, including any differences in regional muscle distribution, have received limited attention. One investigation of collegiate-level sprinters, hurdlers, and jumpers (19) reported that although males had a greater total lower body muscle volume relative to body mass than females, no differences in muscle volumes were noted between males and females when normalized to the product of height and mass (19). We are aware of only one other study that documented the sex differences in the volume of muscle groups between male and female sprinters, with males found to have larger absolute muscle volumes of three thigh muscle groups (quadriceps, hamstrings, and adductors), but only one muscle group (hamstrings) when volume was normalized to body size (20). Notably, this study did not assess volume of the hip or ankle extensor or flexor muscles, although the hip joint muscles previously highlighted as being particularly important for sprint performance among both males and females [e.g., correlation of hip extensor volume per kilogram body mass and performance *r* = −0.560 (males; 12) and *r* = −0.577 (females; 11)]. In addition, neither of the above studies included elite-level sprinters, and both utilized low subject numbers (<9 per sex group; 19, 20). Thus, it appears that no comprehensive comparison of muscle volumes between male and female sprinters currently exists, particularly between elite populations. Moreover, to date no study has compared the proportional size of muscles/muscle groups between sexes (i.e., the size of one muscle compared with total leg muscle volume) to provide an index of lower limb skeletal muscle distribution. Therefore, whether the lower limb skeletal muscle distribution of male and female sprinters is similar, perhaps representing a common selected phenotype, remains unknown. Examining the similarity/distinctiveness of male sprinters to both female sprinters and male controls may reveal if sex or sprint status is the stronger determinant of skeletal muscle distribution.

Whilst careful comparison of male and female sprinters of equivalent relative performance standard (e.g., elite males vs. elite females) may highlight the nature of any differences between these groups, such a comparison cannot isolate if muscle volumes are a cause of the sex difference in performance or purely a coincidental consequence of sex. In fact, it has been suggested that the performance difference between males and females could be a result of greater stature [associated with longer limb lengths (21)], lower relative fat mass, and overall muscularity or the muscularity of specific muscle groups (1, 20, 22). To delineate the importance of these different sex-based characteristics (stature, percentage body fat, muscle volume), for performance it may be insightful to compare elite female sprinters not only to elite male sprinters but also to a group of male sprinters that are performance matched with elite females (i.e., of the same absolute performance standard). This may reveal if differences in putative performance determinants (stature, percentage body fat, total muscle volume, etc.) are associated with sex (i.e., higher in both male groups) or performance (i.e., higher only in the elite males and similar in elite females and performance-matched males) and thus provide insight into the cause of faster sprint times.

Therefore, the aim of this study was to investigate the differences in lower body muscle volume (absolute, relative to body mass, and proportional to total muscle volume) between male and female sprinters, specifically by *1*) comparing two large groups of male and female sprinters; *2*) comparing elite female sprinters with both elite male sprinters and also with performance-matched male sprinters; *3*) assessing if the distribution of muscle volume in elite male sprinters was more distinct from either untrained male controls or elite female sprinters. This study used data drawn from a larger project from our laboratory examining sprint athlete muscle morphology (11, 12). It was hypothesized that *1*) male sprinters would display greater absolute and relative volumes than female sprinters particularly of the hip extensor and flexor muscle groups; *2*) absolute and relative muscle volumes of elite female sprinters would be similar to performance-matched male sprinters, but smaller than elite male sprinters; *3*) elite male sprinters would be more similar to elite female sprinters in their lower body muscle volume distribution than to a group of untrained male controls.

## METHODS

### Participants and Sprint Performance

Thirty-one male (means ± SD; age 23 ± 3 yr, body mass 77.2 ± 8.2 kg, height 1.78 ± 0.06 m) and 22 female (age 24 ± 4 yr, body mass 63.8 ± 6.4 kg, height 1.68 ± 0.06 m) sprinters without minor injury in the previous 4 wk or major injury in the previous 6 mo volunteered to participate and gave informed consent to take part in this study. Participants in the sprint cohorts were required to have a season’s best 100 m sprint time of ≤11.50 s and ≤12.90 s for males and females, respectively, or equivalent times for 60 m/200 m based on International Association of Athletics Federations (IAAF) points, and to have completed at least one season of high-intensity sprint-specific training. Season’s best times were taken from the national governing body database (www.thepowerof10.info) of electronically timed races with wind readings (<2.0 m·s^−1^) during the corresponding calendar year in which data collection took place. Sprint race times were converted to IAAF points, a classification system that permits comparison between athletic events, and each athlete’s maximum points in any of the sprint distances (60, 100, 200 m) was taken as their performance measure and converted back into 100 m season’s best equivalent (SBE_100_) or personal best equivalent (PBE_100_). For each individual, SBE_100_ was also expressed as a percentage of 100 m world record (%WR) to allow for direct performance standard comparisons between sexes. Elite male (EM; *n* = 5) and elite female (EF; *n* = 5) sprint groups were determined by participants who had attained a season’s best British Athletics qualification time for the European Outdoor Championships 2018 (i.e., 10.25 s for males and 11.35 s for females) (23). A group of performance-matched male sprinters with comparable absolute race performance times to the elite female group (PMM_EF_; *n* = 6) were selected from among the full cohort of male sprinters. The data on 11 untrained unathletic males (UM; 26 ± 3 yr, 75.2 ± 5.6 kg, 1.80 ± 0.08 m) were used as a comparator for the third study aim (distinctiveness of EM muscle distribution compared with EF and UM) and were drawn from our previous investigation (12). Ethical approval was granted by the Loughborough University Ethics Approvals (Human Participants) Sub-Committee.

### Study Overview

This cross-sectional study compared between-group measurements taken during one session of MRI to assess muscle volume. Due to limitations in scheduling and practicalities of working with athletes, it was not feasible to control for measurement time of day; however, all sessions were scheduled following a rest or light training day, and participants were instructed to arrive relaxed, having followed habitual daily activity and dietary behaviors, before sitting quietly for 15 min before their MRI scan.

### Anthropometry

Body mass was measured using a calibrated ADAM C-150 weighing scale (ADAM Equipment, Oxford, CT), and stature was measured using a wall-mounted stadiometer (Holtain Ltd., Crymych, UK). Skinfold thickness was measured at eight sites (bicep, tricep, subscapular, iliac crest, supraspinale, abdominal, thigh, and calf) using Harpenden skinfold calipers (British Indicators Ltd., Wolverhampton, UK), and the average of two measurements at each site was recorded. All anthropometric measures were done by the same investigator, and in accordance with the International Society for the Advancement of Kinanthropometry guidelines (24). The sum of four skinfolds (biceps, triceps, subscapular, and iliac crest) was used to calculate individual body density using the appropriate formula according to age (16–19 or 20–29 yr) and sex (male or female) (25), and percentage body fat estimated using the Siri (26) equation. Fat-free mass was derived from the percentage body fat and body mass values.

### Muscle Volume with Magnetic Resonance Imaging

T1-weighted axial magnetic resonance (MR) images of the abdomen, thigh, and shank were obtained with a 3 T scanner (Discovery MR750w; GE Healthcare, Chicago, IL) with a receiver 8-channel whole body coil. Images (time of repetition 600 ms, time of echo 8 ms, field of view 450 × 450 mm, image matrix 320 × 320, pixel size 1.4 × 1.4 mm, slice thickness 5 mm, interslice gap 5 mm) were obtained from the 12th thoracic vertebra to the calcaneus capturing both legs in five overlapping blocks. Participants were scanned while in the supine position with arms folded across the chest, with hip and knee joints extended and the ankle joint at ∼90°. Oil filled capsules were placed in equal segments on the right leg of each participant during scanning, to facilitate alignment between the blocks during analysis.

Six individual investigators carried out analysis of the MR images, with each investigator analyzing the same muscles/compartments for the entire cohort, blinded to participant identity. For muscles that were 200 mm or longer, every other MR image (i.e., 20 mm between the center of the measured images) was manually segmented, starting from the most proximal image in which that muscle/compartment appeared to assess cross-sectional area (CSA) and subsequently derive volumes of 23 lower limb muscles/compartments using a public domain DICOM software (Horos, version 2.2.0, https://horosproject.org/). Every MR image (i.e., 10 mm between center of measure images) was analyzed for muscles shorter than 200 mm. Fully analyzed images for each participant (i.e., all 23 muscles/compartments) were then checked and quality assured for accuracy by a single investigator (R.M.), paying particular attention to errors and overlaps between adjacent muscle cross sections. The analyzed muscles/compartments were iliopsoas (psoas major and iliacus combined); sartorius; tensor fasciae latae (TFL); adductor magnus; gracilis; gluteus maximus; gluteus medius; gluteus minimus; rectus femoris; vastus lateralis, medialis, and intermedius; semimembranosus; semitendinosus; biceps femoris long and short heads; popliteus; lateral and medial gastrocnemius; soleus; and the anterior, lateral, and deep posterior compartments of the shank. The shank compartments were the combined volume of the following muscles: tibialis anterior, extensor digitorum longus, and extensor hallucis longus (anterior); peroneus longus and brevis (lateral); plantaris, tibialis posterior, flexor digitorum longus, and flexor hallucis longus (deep posterior). The volume of five functional muscle groups was calculated as the sum of the following muscles: hip extensors (HE; gluteus maximus, adductor magnus, biceps femoris long head, semimembranosus, and semitendinosus); hip flexors (HF; iliopsoas, rectus femoris, sartorius, and TFL); knee extensors (KE; rectus femoris, vastus intermedius, medialis, and lateralis); knee flexors (KF; gracilis, biceps femoris long and short head, semimembranosus, semitendinosus, sartorius, popliteus, and medial and lateral gastrocnemius); and plantarflexors (PF; medial and lateral gastrocnemius and soleus).

The volume of each muscle (V*_m_*) was calculated using previously outlined methods (15):

$${\text{V}}_{m}=\overset{n-1}{\underset{i=1}{\sum }}\frac{h}{2}\left(Am_{i}+Am_{i+1}\right)$$

where *Am* represents the muscle cross-sectional area calculated from each image, *i* is the image number, *n* is the total number of images, and *h* is the distance between images (20 mm). In addition to absolute muscle volume (cm^3^), muscle volume was also expressed relative to body mass (cm^3^·kg^−1^). To understand muscle volume distribution and to remove the confounding effect of body size, the volume of each individual muscle/compartment was also expressed as a percentage of total unilateral lower body muscle volume (%TMV) calculated using the following equation:

$$\%\text{TMV}=\left(\frac{\text{V}m_{\chi }}{\text{V}m_{\text{All}}}\right)\times 100$$

where V*m_χ_* is the absolute volume of the individual muscle/compartment, and V*m*_All_ is the summed absolute volume of all 23 muscles/compartments. In our laboratory, we have found the MRI measurement of muscle volume (including image collection and analysis) for various muscle groups to be highly reliable with a coefficient of variation of ∼1% (27, 28).

### Statistical Analysis

Muscle volume measurements assessed on both legs were averaged for each participant to provide unilateral criterion values. Muscle volume values were expressed in absolute, relative to body mass, and as %TMV, and data are presented as means ± standard deviation (SD), unless otherwise stated. The Shapiro–Wilk test was used to assess the normality of distribution and revealed that >92% of the variables were normally distributed, in which case parametric statistical tests were used to provide a consistent approach. An independent-samples *t* test was used to assess differences between male and female cohorts for muscle volume and anthropometry, and a Bonferroni–Holm correction for multiple comparison was applied to family-wise variables. One-way ANOVA and subsequent Bonferroni post hoc analysis were used to assess differences between subgroups (EF, EM, and PMM_EF_) for muscle volume and anthropometry. The between-group differences (i.e., EM vs. EF, EM vs. UM) in %TMV for all 23 individual muscles/compartments were calculated and were not normally distributed. Subsequently, to assess if the muscle distribution of EM was more distinct from UM than EF, a Mann–Whitney *U* test was used to compare these intergroup differences (i.e., the differences between EM and EF were compared with the differences between EM and UM). Statistical significance was set at *P* < 0.05. All statistical procedures were performed with IBM SPSS Statistics Version 25 (IBM Corp., New York, NY).

## RESULTS

### Sprint Performance and Training History

The full cohort of male sprinters were 0.99 s (9%) faster for SBE_100_ and 0.98 s (8%) faster for PBE_100_ (both *P* = 0.01) than the whole cohort of female sprinters; however, the relative performance standard of the groups (i.e., IAAF points and %WR) were equivalent (*P* ≥ 0.709; Table 1). There were also no differences in sprint or resistance training history (*P* = 0.122 and *P* = 0.70, respectively) between the groups.

**Table 1.** Race performance, training status, and anthropometric characteristics

	Full Cohorts		Subgroups		
	Female Sprinters	Male Sprinters	EF	EM	PMM_EF_
Sprint race performance
SBE_100_, s	11.68 ± 0.47	10.69 ± 0.38**	11.16 ± 0.06‡‡	10.10 ± 0.07†† ^̂	11.17 ± 0.19‡‡
PBE_100_, s	11.56 ± 0.35	10.58 ± 0.36**	11.14 ± 0.04‡‡	9.99 ± 0.07†† ^̂	11.04 ± 0.14‡‡
IAAF points	1,057 ± 94	985 ± 118	1,165 ± 12̂̂	1,174 ± 26̂̂	839 ± 53†† ‡‡
SBE_100_ (% of world record)	111 ± 4	112 ± 4	106 ± 1̂̂	105 ± 1̂̂	117 ± 2†† ‡‡
Training history
Sprint training duration, yr	7 ± 4	6 ± 3	8.2 ± 2.9	9.1 ± 2.9	5.0 ± 2.7
Resistance training duration, yr	6 ± 4	4 ± 3	6.8 ± 2.8‡‡ ^	7.8 ± 2.4̂̂	2.6 ± 0.9† ‡‡
Anthropometrics
Age, yr	24 ± 4	23 ± 3	25.4 ± 4.0‡	27.4 ± 4.1	21.8 ± 1.8
Height, m	1.68 ± 0.06	1.78 ± 0.06*	1.68 ± 0.05‡‡ ^	1.83 ± 0.06††	1.77 ± 0.04†
Body mass, kg	63.8 ± 6.4	77.2 ± 8.2**	67.7 ± 6.0‡‡	86.4 ± 6.7†† ^̂	72.7 ± 3.8‡‡
Body mass index, kg·m^–2^	22.6 ± 2.0	24.3 ± 2.3*	24.0 ± 2.9	26.1 ± 3.4	23.3 ± 2.1
Sum of 8 skinfold, mm	67.4 ± 15.3	51.0 ± 13.8*	61.0 ± 9.7‡	39.4 ± 3.6†	55.8 ± 14.4
Body fat percentage, %	19.7 ± 3.3	10.8 ± 3.1**	18.5 ± 4.1‡‡ ^̂	8.3 ± 1.2††	11.8 ± 2.7††
Fat free mass, kg	52.1 ± 5.1	68.7 ± 7.9**	55.1 ± 5.2‡‡ ^	79.8 ± 6.1†† ^̂	64.1 ± 3.2† ‡‡

Data are means ± SD. Data are given for the full cohorts of male (*n* = 31) and female (*n* = 22) sprinters, and subgroups of elite female (EF; *n* = 5), elite male (EM; *n* = 5), and performance-matched male sprinters (to elite females; PMM_EF_; *n* = 6). SBE_100_, 100-m season’s best equivalent; PBE_100_, 100-m personal best equivalent; IAAF, International Association of Athletics Federations. Significantly different from female sprinters: *P ≤ 0.05 and **P ≤ 0.01. Significantly different from EF: †*P* ≤ 0.05 and ††*P* ≤ 0.01. Significantly different from EM ‡*P* ≤ 0.05 and ‡‡*P* ≤ 0.01. Significantly different from PMM_EF_ ^*P* ≤ 0.05 and ^̂*P* ≤ 0.01.

EF were 1.06 s (10%) slower for SBE_100_ and 1.15 s (12%) slower for PBE_100_ (both *P* < 0.0001) when compared with EM, but there were no differences in IAAF points, %WR, sprint, or resistance training history between these subgroups (*P* ≥ 0.816; Table 1). EF and PMM_EF_ had similar absolute performance times (SBE_100_: 11.16 vs. 11.17 s; *P* = 1.000), but as expected EF had a higher relative performance standard when compared with PMM_EF_ (IAAF points 1,165 vs. 839, *P* < 0.0001; %WR 106% vs. 117%; *P* < 0.001). EF also had a longer resistance training history (*P* = 0.018) but similar sprint training history than PMM_EF_ (*P* = 0.253).

### Anthropometrics

When considering the full cohorts, male sprinters were taller and heavier than female sprinters, had a greater body mass index (BMI), were leaner, and had greater fat-free mass than female sprinters (all *P* ≤ 0.008). EF were also shorter in stature, lighter, had a greater body fat percentage, and had less fat-free mass than EM (all *P* ≤ 0.037); however, no differences in BMI were observed between these groups (*P* = 0.757). Compared with PMM_EF_, EF were smaller in stature, had a greater body fat percentage, and less fat-free mass (all *P* ≤ 0.025), as well as having a tendency for less body mass (*P* = 0.068).

### Comparison of Muscle Volumes between the Whole Male versus Female Sprint Cohorts

**Figure 1.**
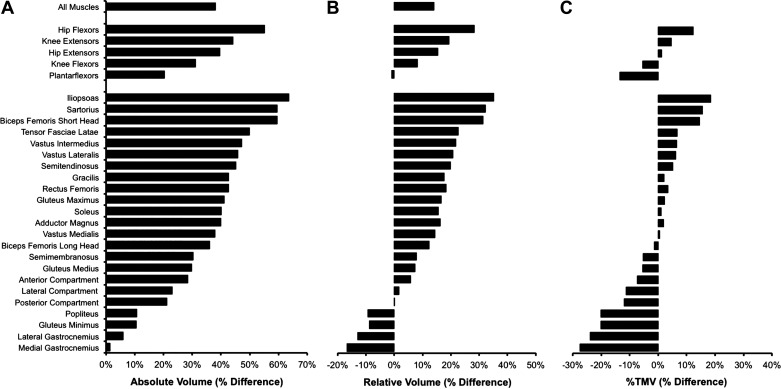
Percentage differences in muscle volumes between male (*n* = 31) and female (*n* = 22) sprinters expressed in absolute (*A*, cm^3^), relative to body mass (*B*, cm^3^·kg^−1^), and percentage of total unilateral muscle volume (*C*, %TMV) terms for all muscles, 5 functional muscle groups, and 23 individual muscles/compartments. A positive value indicates greater volume of male sprinters. The functional muscle groups and individual muscles are ordered according to the magnitude of the percentage differences for absolute muscle volume.

Absolute muscle volumes were larger in male versus female sprinters for total unilateral muscle volume by 38% (*P* = 0.008), all 5 functional muscle groups (HF +55%, KE +44%, HE +40%, KF +31%, PF +20%; all *P* ≤ 0.05), and 20 out of 23 individual lower body muscles/compartments (*P* < 0.010; Table 2; Fig. 1*A*).

**Table 2.** Absolute, relative, and percentage of total muscle volume of the full cohorts of male and female sprinters

Muscle Group/Muscle	Absolute Muscle Volume, cm^3^		Relative Muscle Volume, cm^3^·kg^–1^		Percentage of Total Unilateral Muscle Volume (%TMV)	
	Female Sprinters	Male Sprinters	Female Sprinters	Male Sprinters	Female Sprinters	Male Sprinters
Total unilateral muscle volume	6,893 ± 930	9,513 ± 1,449**	107.9 ± 8.1	123.1 ± 10.2**		
Hip flexors	879 ± 141	1,363 ± 239**	13.8 ± 1.6	17.6 ± 2.2**	12.7 ± 1.0	14.3 ± 1.1**
Hip extensors	2,283 ± 379	3,186 ± 559*	35.7 ± 3.7	41.2 ± 4.3*	33.0 ± 2.1	33.4 ± 1.9
Knee flexors	1,473 ± 187	1,930 ± 328*	23.1 ± 2.0	25.0 ± 2.7*	21.4 ± 1.5	20.3 ± 1.2*
Knee extensors	1,895 ± 346	2,730 ± 450*	29.6 ± 4.0	35.4 ± 4.2*	27.4 ± 2.4	28.7 ± 1.8
Plantarflexors	806 ± 145	970 ± 169*	12.7 ± 2.0	12.5 ± 1.4	11.8 ± 2.2	10.2 ± 1.1*
Iliopsoas	386 ± 58	631 ± 104**	6.1 ± 0.7	8.2 ± 1.0**	5.6 ± 0.5	6.7 ± 0.6**
Sartorius	141 ± 38	224 ± 61**	2.2 ± 0.5	2.9 ± 0.7**	2.0 ± 0.4	2.3 ± 0.4
Tensor fasciae latae	63 ± 18	94 ± 33**	1.0 ± 0.3	1.2 ± 0.3	0.9 ± 0.2	1.0 ± 0.2
Adductor magnus	618 ± 116	864 ± 148**	9.6 ± 1.2	11.2 ± 1.5**	8.9 ± 0.9	9.1 ± 1.1
Gracilis	104 ± 22	148 ± 39**	1.6 ± 0.3	1.9 ± 0.4	1.5 ± 0.3	1.5 ± 0.3
Gluteus maximus	953 ± 190	1,344 ± 303**	14.9 ± 2.1	17.3 ± 2.5**	13.7 ± 1.4	14.0 ± 1.4
Gluteus medius	316 ± 52	410 ± 72**	5.0 ± 0.7	5.3 ± 0.8	4.6 ± 0.5	4.3 ± 0.7
Gluteus minimus	157 ± 33	173 ± 38	2.5 ± 0.5	2.2 ± 0.4	2.3 ± 0.4	1.8 ± 0.4**
Rectus femoris	290 ± 55	413 ± 78**	4.5 ± 0.7	5.4 ± 0.9**	4.2 ± 0.5	4.3 ± 0.5
Vastus lateralis	657 ± 138	958 ± 175**	10.3 ± 1.6	12.4 ± 1.6**	9.5 ± 1.1	10.1 ± 0.9
Vastus intermedius	555 ± 106	817 ± 152**	8.7 ± 1.3	10.6 ± 1.6**	8.1 ± 1.0	8.6 ± 0.9
Vastus medialis	393 ± 75	542 ± 93**	6.1 ± 0.9	7.0 ± 0.9**	5.7 ± 0.5	5.7 ± 0.5
Semimembranosus	255 ± 46	332 ± 60**	4.0 ± 0.6	4.3 ± 0.6	3.7 ± 0.5	3.5 ± 0.5
Semitendinosus	252 ± 48	366 ± 85**	3.9 ± 0.6	4.7 ± 0.8**	3.6 ± 0.4	3.8 ± 0.5
Biceps femoris long head	205 ± 38	279 ± 52**	3.2 ± 0.5	3.6 ± 0.5	3.0 ± 0.4	2.9 ± 0.4
Biceps femoris short head	86 ± 17	137 ± 35**	1.3 ± 0.2	1.8 ± 0.4**	1.2 ± 0.2	1.4 ± 0.3
Popliteus	16 ± 4	18 ± 6	0.3 ± 0.1	0.2 ± 0.1	0.2 ± 0.0	0.2 ± 0.0**
Lateral gastrocnemius	166 ± 30	175 ± 38	2.6 ± 0.5	2.3 ± 0.4	2.4 ± 0.4	1.8 ± 0.3**
Medial gastrocnemius	264 ± 67	268 ± 57	4.2 ± 1.0	3.5 ± 0.6**	3.9 ± 1.0	2.8 ± 0.4**
Soleus	376 ± 70	526 ± 93**	5.9 ± 0.9	6.8 ± 0.8**	5.5 ± 1.1	5.6 ± 0.7
Anterior compartment	216 ± 32	278 ± 49**	3.4 ± 0.5	3.6 ± 0.5	3.2 ± 0.4	2.9 ± 0.4
Lateral compartment	129 ± 20	159 ± 40**	2.0 ± 0.3	2.1 ± 0.5	1.9 ± 0.3	1.7 ± 0.3
Posterior compartment	292 ± 69	354 ± 74**	4.6 ± 0.9	4.6 ± 0.8	4.2 ± 0.7	3.7 ± 0.6

Data are given for all muscles (%TMV), 5 functional muscle groups, and 23 individual muscles/compartments for the full cohorts of male (*n* = 31) and female (*n* = 22) sprinters. Muscle volume data are presented as group means ± SD, with individual measurements the average of both sides/legs (i.e., unilateral). All muscles are the sum of muscle volumes from all the muscles/compartments listed. Significantly different from female sprinters: *P ≤ 0.05 and **P ≤ 0.01.

Relative to body mass, total unilateral muscle volume was 14% larger in male versus female sprinters (*P* = 0.010; Fig. 1*B*), along with four of the five functional muscle groups (HF +28%, KE +19%, HE +15%, KF +8%; all *P* ≤ 0.025), but not the plantarflexors (*P* = 0.809). Relative to body mass, 11 individual muscles/compartments were greater in male sprinters (iliopsoas +35%, sartorius +32%, biceps femoris short head +31%, vastus intermedius +22%, vastus lateralis +21%, semitendinosus +20%, rectus femoris +18%, gluteus maximus +17%, adductor magnus +16%, soleus +16%, and vastus medialis +14%; all *P* ≤ 0.004), whereas the medial gastrocnemius was smaller in male sprinters versus female sprinters (−17%; *P* = 0.004).

When the volume of functional muscle groups and individual muscles/compartments was expressed as a percentage of total unilateral muscle volume (%TMV), the HF were a greater proportion of lower body muscle volume in male sprinters than in female sprinters (14.3 vs. 12.7% TMV; *P* = 0.010), whereas the KF and PF were proportionally smaller in males than in females (20.3 vs. 21.4% TMV and 10.2 vs. 11.8% TMV, respectively; both *P* ≤ 0.017; Fig. 1*C*). The iliopsoas was larger relative to total muscle volume in male versus female sprinters (6.7 vs. 5.6% TMV; *P* = 0.002), whereas four muscles were a smaller percentage of total volume in males versus females (gluteus minimus 1.8 vs. 2.3% TMV; popliteus 0.23 vs. 0.19% TMV; medial gastrocnemius 2.8 vs. 3.9% TMV; and lateral gastrocnemius 1.8 vs. 2.4% TMV; all *P* ≤ 0.003).

### Muscle Volume Comparison of Subgroups: Elite Female Sprinters, Performance-Matched Male Sprinters, and Elite Male Sprinters

**Figure 2.**
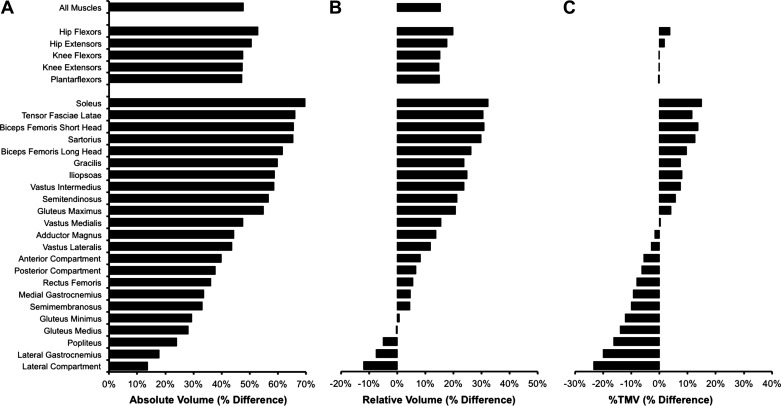
Percentage differences in muscle volumes between elite male (*n* = 5) and elite female (*n* = 5) sprinters expressed in absolute (*A*, cm^3^), relative to body mass (*B*, cm^3^·kg^−1^), and percentage of total unilateral muscle volume (*C*, %TMV) terms for all muscles, 5 functional muscle groups, and 23 individual muscles/compartments. A positive value indicates greater volume of male sprinters. The functional muscle groups and individual muscles are ordered according to the magnitude of the percentage differences for absolute muscle volume.

**Figure 3.**
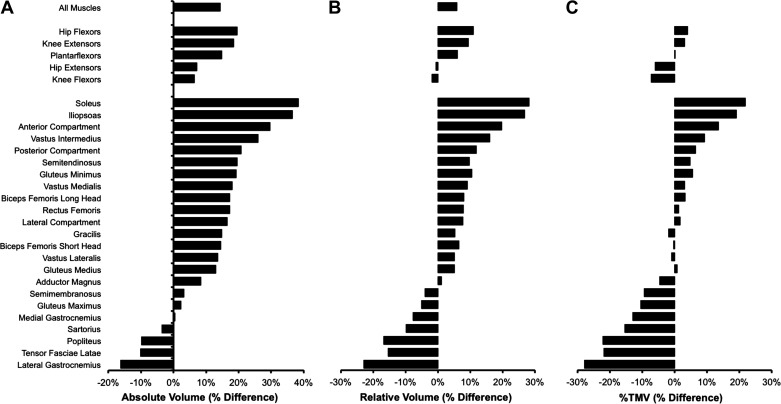
Percentage differences in muscle volumes between elite female (*n* = 5) sprinters and performance-matched male sprinters (*n* = 6) expressed in absolute (*A*, cm^3^), relative to body mass (*B*, cm^3^·kg^−1^), and percentage of total unilateral muscle volume (*C*, %TMV) terms for all muscles, 5 functional muscle groups, and 23 individual muscles/compartments. A positive value indicates greater volume of performance-matched male sprinters. The functional muscle groups and individual muscles are ordered according to the magnitude of the percentage differences for absolute muscle volume.

When considering absolute muscle volumes, EM had greater total unilateral volume compared with both EF (+48%, *P* < 0.0001; Table 3) and PMM_EF_ (+29%, *P* = 0.005). In addition, EM had greater volumes of all five functional muscle groups (HF +53%, HE +50%, KF +47%, KE +47% PF +47%; all *P* ≤ 0.005) compared with EF (Fig. 2), and of four functional muscle groups (HF +28%, HE +40%, KF 39%, PF +28%; all *P* ≤ 0.043) compared with PMM_EF_. EM also displayed greater absolute volumes in 14 and 9, out of 23, individual muscles/compartments compared with EF and PMM_EF_, respectively (all *P* ≤ 0.047). EF were similar to PMM_EF_ for total absolute unilateral muscle volume and the volumes of all five functional muscle groups (*P* ≥ 0.397), and only one individual muscle (iliopsoas −27%, *P* = 0.032) was smaller in EF versus PMM_EF_ (Fig. 3).

**Table 3.** Absolute and relative muscle volume of all muscles of elite male, elite females, and performance-matched male (to elite female) sprinters

Muscle Group/Muscle	Absolute Muscle Volume, cm^3^			Relative Muscle Volume, cm^3^·kg^–1^		
	EM	PMM_EF_	EF	EM	PMM_EF_	EF
Total unilateral muscle volume	11,323 ± 1,328** ‡	8,764 ± 1,112††	7,665 ± 585††	131.3 ± 6.8*	120.5 ± 12.8	113.8 ± 6.8†
Hip flexors	1,620 ± 200** ‡	1,266 ± 270†	1,059 ± 87††	18.8 ± 1.8	17.4 ± 3.6	15.7 ± 0.9
Hip extensors	4,002 ± 489** ‡‡	2,849 ± 252††	2,660 ± 209††	46.4 ± 2.9** ‡‡	39.2 ± 2.6††	39.4 ± 0.8††
Knee flexors	2,304 ± 178** ‡‡	1,662 ± 200††	1,562 ± 79††	26.8 ± 0.8* ‡‡	22.8 ± 2.1††	23.2 ± 1.6†
Knee extensors	3,218 ± 400**	2,588 ± 519	2,184 ± 253††	37.3 ± 2.5	35.5 ± 6.3	32.5 ± 4.1
Plantarflexors	1,112 ± 181** ‡	867 ± 116†	756 ± 56††	12.9 ± 1.8	11.9 ± 1.2	11.2 ± 0.8
Iliopsoas	702 ± 97**	604 ± 107*	443 ± 49†† ‡	8.2 ± 1.1	8.3 ± 1.3*	6.5 ± 0.3‡
Sartorius	306 ± 46** ‡‡	179 ± 60††	185 ± 37††	3.6 ± 0.4‡	2.5 ± 0.9†	2.7 ± 0.4
Tensor fasciae latae	135 ± 41* ‡	73 ± 29†	81 ± 24	1.6 ± 0.4	1.0 ± 0.4	1.2 ± 0.3
Adductor magnus	1,056 ± 83** ‡‡	792 ± 109††	732 ± 100††	12.3 ± 1.0	10.9 ± 1.6	10.8 ± 0.9
Gracilis	180 ± 37*	130 ± 38	113 ± 9†	2.1 ± 0.4	1.8 ± 0.5	1.7 ± 0.3
gluteus maximus	1,797 ± 376** ‡‡	1,188 ± 183††	1,161 ± 128††	20.8 ± 3.1‡	16.3 ± 2.1†	17.2 ± 1.1
Gluteus medius	434 ± 92	383 ± 64	339 ± 53	5.0 ± 0.7	5.3 ± 0.9	5.0 ± 0.6
Gluteus minimus	192 ± 46	177 ± 52	148 ± 38	2.2 ± 0.5	2.4 ± 0.7	2.2 ± 0.6
Rectus femoris	476 ± 45	410 ± 108	350 ± 39	5.5 ± 0.4	5.6 ± 1.5	5.2 ± 0.9
Vastus lateralis	1,132 ± 180	894 ± 165	788 ± 113†	13.1 ± 1.1	12.3 ± 2.0	11.7 ± 1.6
Vastus intermedius	962 ± 145**	765 ± 190	607 ± 74††	11.2 ± 1.3	10.5 ± 2.3	9.0 ± 1.2
Vastus medialis	649 ± 97**	519 ± 93	440 ± 62††	7.5 ± 0.9	7.1 ± 1.0	6.5 ± 0.8
Semimembranosus	359 ± 60	278 ± 26	270 ± 65	4.2 ± 0.6	3.8 ± 0.3	4.0 ± 0.8
Semitendinosus	449 ± 70** ‡	343 ± 53†	287 ± 51††	5.2 ± 0.5	4.7 ± 0.6	4.3 ± 0.9
Biceps femoris long head	340 ± 31** ‡‡	247 ± 29††	210 ± 26††	4.0 ± 0.3*	3.4 ± 0.3	3.1 ± 0.5†
Biceps femoris short head	167 ± 26** ‡	115 ± 30†	101 ± 17††	1.9 ± 0.3	1.6 ± 0.4	1.5 ± 0.2
Popliteus	23 ± 5	17 ± 7	19 ± 3	0.3 ± 0.1	0.2 ± 0.1	0.3 ± 0.1
Lateral gastrocnemius	202 ± 34‡	144 ± 30†	172 ± 27	2.4 ± 0.5	2.0 ± 0.4	2.6 ± 0.5
Medial gastrocnemius	300 ± 38* ‡	226 ± 56†	225 ± 25†	3.5 ± 0.4	3.1 ± 0.7	3.3 ± 0.4
Soleus	610 ± 137**	497 ± 40	359 ± 64†	7.1 ± 1.3*	6.8 ± 0.3*	5.3 ± 0.8† ‡
Anterior compartment	302 ± 59*	280 ± 45	216 ± 28†	3.5 ± 0.5	3.9 ± 0.6	3.2 ± 0.5
Lateral compartment	147 ± 32	150 ± 24	129 ± 17	1.7 ± 0.3	2.1 ± 0.3	1.9 ± 0.3
Posterior compartment	401 ± 76	352 ± 77	291 ± 91	4.6 ± 0.6	4.9 ± 1.1	4.3 ± 1.4

Data are given for elite male (EM, *n* = 5), performance-matched male (to elite female; PMM_EF_, *n* = 6), and elite female (EF, *n* = 5) sprinters for all muscles, 5 functional muscle groups, and 23 individual muscles/compartments. Muscle volume data are presented as group means ± SD, with individual measurements the average of both sides/legs (i.e., unilateral). Total unilateral muscle volume is the sum of muscle volumes from all the muscles/compartments listed. Significantly different from elite female sprinters: *P ≤ 0.05 and **P ≤ 0.01. Significantly different from elite male sprinters: †*P* ≤ 0.05 and ††*P* ≤ 0.01. Significantly different from performance-matched male sprinters: ‡*P* ≤ 0.05 and ‡‡*P* ≤ 0.01.

Relative to body mass, total unilateral muscle volume was greater in EM versus EF (+15%, *P* = 0.037; Fig. 2*B*), but not EM versus PMM_EF_ (+9%, *P* = 0.254; Table 3). Two functional muscle groups were larger in EM compared with both EF (HE +18%, KF +15%; both *P* ≤ 0.013) and PMM_EF_ (HE +18%, KF +17%; both *P* ≤ 0.004), and only two individual muscles were larger in EM compared with both EF (biceps femoris long head, +26%, *P* = 0.016; soleus, +32%, *P* = 0.022) and PMM_EF_ (gluteus maximus, +27%; sartorius, +44%) relative to body mass. EF and PMM_EF_ had similar total unilateral muscle volume and volume of all five functional muscle groups (*P* ≥ 0.803), relative to body mass, although EF had two individual muscles that were smaller than PMM_EF_ relative to body mass (soleus −22%, iliopsoas −21%; both *P* ≤ 0.044).

When muscle volumes were expressed as a percentage of total muscle volume, no differences were observed between subgroups for any functional muscle group (EM vs. EF, *P* ≥ 0.999; EF vs. PMM_EF_, *P* ≥ 0.056; Table 4). With regard to individual muscles/compartments, the lateral compartment of the shank was smaller in EM compared with EF and PMM_EF_ (1.3 vs. 1.7% and 1.7% TMV, respectively; both *P* ≤ 0.018). EM had greater proportional volume of the gluteus maximus (15.7 vs. 13.5% TMV, respectively; *P* = 0.016) compared with PMM_EF_. As a percentage of total lower body muscle volume, the iliopsoas was smaller (5.8 vs. 6.9% TMV, respectively; *P* = 0.027), and the lateral gastrocnemius greater (2.3 vs. 1.6% TMV, respectively; *P* = 0.045) in EM versus PMM_EF._

**Table 4.** Percentage of total muscle volume of elite male, elite female, and performance-matched male (to elite female) sprinters

Muscle Group/Muscle	% Total Muscle Volume		
	EM	PMM_EF_	EF
Hip flexors	14.3 ± 1.0	14.4 ± 1.8	13.8 ± 0.3
Hip extensors	35.3 ± 0.9	32.6 ± 1.8	34.7 ± 2.0
Knee flexors	20.4 ± 1.0	19.0 ± 0.8	20.5 ± 1.6
Knee extensors	28.4 ± 0.8	29.3 ± 2.4	28.5 ± 2.1
Plantarflexors	9.9 ± 1.5	9.9 ± 0.8	9.9 ± 1.0
Iliopsoas	6.2 ± 0.8	6.9 ± 0.5*	5.8 ± 0.5
Sartorius	2.7 ± 0.2	2.0 ± 0.6	2.4 ± 0.3
Tensor fasciae latae	1.2 ± 0.3	0.8 ± 0.3	1.1 ± 0.3
Adductor magnus	9.4 ± 1.1	9.1 ± 1.5	9.6 ± 1.3
Gracilis	1.6 ± 0.3	1.5 ± 0.2	1.5 ± 0.2
Gluteus maximus	15.7 ± 1.6	13.5 ± 0.7†	15.1 ± 0.7
Gluteus medius	3.8 ± 0.4	4.5 ± 1.1	4.4 ± 0.5
Gluteus minimus	1.7 ± 0.3	2.0 ± 0.6	1.9 ± 0.4
Rectus femoris	4.2 ± 0.2	4.6 ± 0.8	4.6 ± 0.6
Vastus lateralis	10.0 ± 0.7	10.2 ± 0.9	10.3 ± 1.0
Vastus intermedius	8.5 ± 0.7	8.6 ± 1.1	7.9 ± 0.7
Vastus medialis	5.7 ± 0.6	5.9 ± 0.5	5.7 ± 0.5
Semimembranosus	3.2 ± 0.5	3.2 ± 0.4	3.5 ± 0.9
Semitendinosus	4.0 ± 0.2	3.9 ± 0.5	3.7 ± 0.6
Biceps femoris long head	3.0 ± 0.3	2.9 ± 0.5	2.8 ± 0.5
Biceps femoris short head	1.5 ± 0.3	1.3 ± 0.2	1.3 ± 0.2
Popliteus	0.2 ± 0.0	0.2 ± 0.1	0.2 ± 0.0
Lateral gastrocnemius	1.8 ± 0.4	1.6 ± 0.1*	2.3 ± 0.5
Medial gastrocnemius	2.7 ± 0.4	2.6 ± 0.5	2.9 ± 0.4
Soleus	5.4 ± 1.0	5.7 ± 0.5	4.7 ± 0.7
Anterior compartment	2.7 ± 0.4	3.2 ± 0.5	2.8 ± 0.3
Lateral compartment	1.3 ± 0.2*	1.7 ± 0.2††	1.7 ± 0.2†
Posterior compartment	3.5 ± 0.5	4.0 ± 0.8	3.8 ± 1.0

Muscle volume data are presented as group means ± SD, with individual measurements the average of both sides/legs (i.e., unilateral). Data are given for 5 functional muscle groups and 23 individual muscles/compartments of elite male (EM, *n* = 5), performance-matched male (to elite female; PMM_EF_, *n* = 6), and elite female (EF, *n* = 5) sprinters. Significantly different from elite female sprinters: *P ≤ 0.05. Significantly different elite male sprinters: †*P* ≤ 0.05 and ††*P* ≤ 0.01.

**Figure 4.**
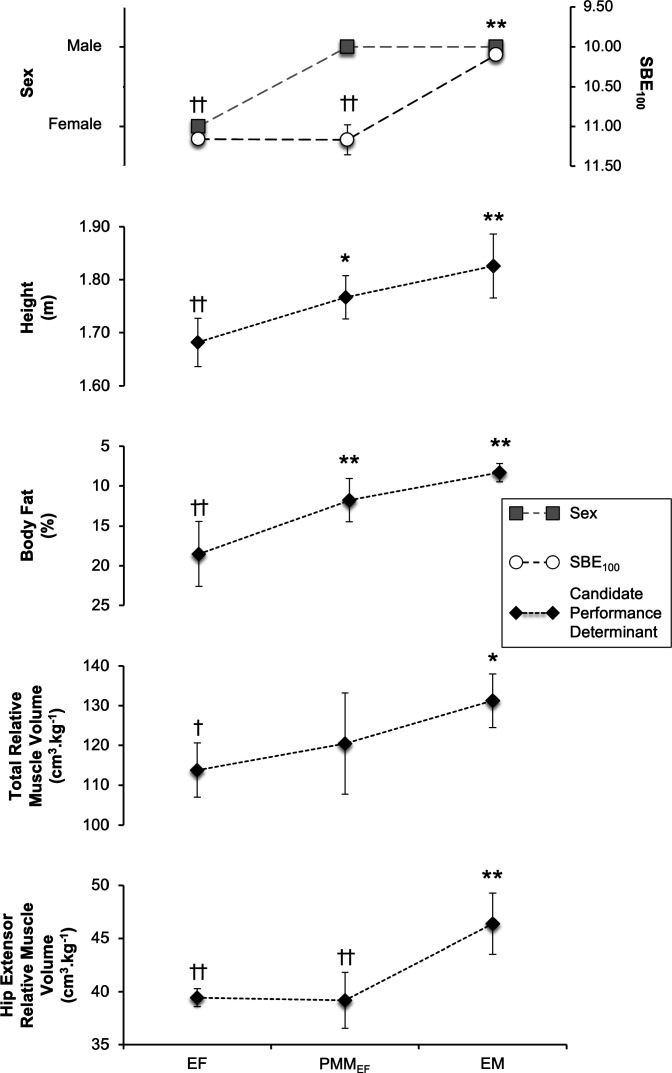
Comparison of SBE_100_, sex, and candidate performance determinants (height, body fat percentage, and relative total unilateral and hip extensor volumes) between elite male (EM, *n* = 5), elite female (EF, *n* = 5), and performance-matched male (to elite female; PMM_EF_, *n* = 6) sprinters. Data are presented as group means ± SD. Although relative hip extensor volume is shown, relative knee flexor volume also differentiated for performance rather than sex (i.e., PMM_EF_ and EF < EM). Significantly different from elite female sprinters: *P ≤ 0.05 and **P ≤ 0.01, significantly different from elite male sprinters †*P* ≤ 0.05 and ††*P* ≤ 0.01. Significance in *top* relates to SBE_100_. SBE_100_, 100-m season’s best equivalent.

Considering the candidate mechanisms for the superior sprint performance of EM versus PMM_EF_ and EF, as opposed to the sex dichotomy (EM and PMM_EF_ vs. EF), PMM_EF_ had greater height and less body fat than EF, while being similar to EM for these traits (Fig. 4), suggesting these traits were in accordance with the sex of PMM_EF_ rather than their performance. Due to the differences in body height and mass between the three subgroups, only relative muscle volumes (cm^3^·kg^−1^) were considered as candidate performance determinants when comparing the three subgroups. For total relative muscle volume PMM_EF_ were not different from either EF or EM. However, for relative volume of the hip extensors and knee flexors PMM_EF_ were significantly lower than EM and similar to EF, and therefore these traits appeared to be in accordance with the performance of PMM_EF_ rather than their sex. No other relative muscle volumes, of either functional muscle groups or individual muscles/compartments, differentiated for performance rather than sex.

### Relationship of Muscle Volume Measures between Subgroups and Comparison of Muscle Volume Distribution

**Figure 5.**
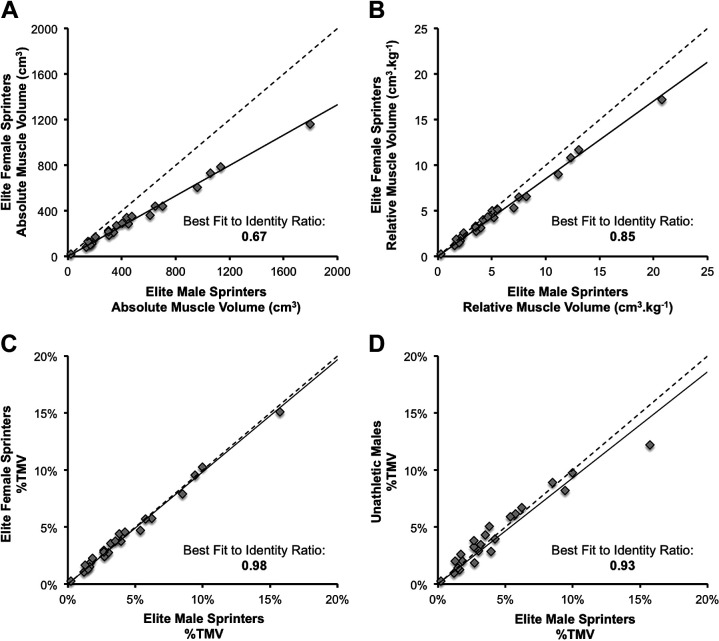
Scatter plots showing the relationship between elite male and female sprinters for the size of 23 individual muscles/compartments (each marker is one muscle/compartment), expressed as absolute muscle volume (cm^3^) (*A*), relative muscle volume (*B*) (cm^3^·kg^−1^), and percentage of total unilateral volume (%TMV) (*C*). For comparison with *C*, *D* also shows the relationship between elite male sprinters and unathletic males for the %TMV of 23 individual muscles/compartments. The dotted line represents the line of identity (i.e., points below the line of identity indicate greater volume for elite male sprinters, and points above the line of identity greater volume for elite female sprinters/unathletic males). The solid line is a linear trend line plotted for all individual muscles/compartments, and the best fit to identity ratio represents the ratio between the slopes of both lines.

As expected, graphical representation of the relationship between EF and EM absolute muscle volumes of all 23 muscles (Fig. 5*A*) indicated a substantial bias toward EF having smaller muscles (i.e., the slope of the line of best fit is 67% of the line of identity). When all 23 muscle volumes were expressed relative to body mass (i.e., cm^3^·kg^−1^; Fig. 5*B*), there was a markedly lower bias toward EF having smaller muscles than EM (the slope of the line of best was 85% of the line of identity). Finally, when all 23 muscle volumes were expressed at %TMV (Fig. 5*C*), the line of best fit was very similar to the line of identity (98% of the slope). The scatter plots reveal that when considering all 23 muscles/compartments together the differences between EF and EM reduced (came closer to identity) with normalization to body mass, and reduced further when expressed as a percentage of total unilateral volume.

Subsequently, to determine if the muscle distribution (%TMV) across the 23 leg muscle/compartments of EM was more similar to EF than to UM, the between-group differences from both these comparisons were compared. The comparison of EM versus UM showed a mean absolute difference of 18.1 ± 12.8%, which was significantly greater than the mean absolute difference between EM versus EF of 9.8 ± 5.7% (*U* = 170.5, *P* = 0.039), suggesting that EM were more similar to EF (Fig. 5*C*) than they were to UM (Fig. 5*D*).

## DISCUSSION

This study compared lower body muscle volumes between two large cohorts of male and female sprinters, as well as comparing elite female sprinters to both elite and performance-matched male sprinters, and finally assessing whether the muscle distribution of elite male sprinters was more similar to/distinct from elite female sprinters or unathletic males. In support of our first hypothesis, although there were some pronounced sex differences in the volume of some muscle groups this was nonuniform and anatomically specific, whereby larger differences were observed in the volumes of the muscle groups of the hip (males +40–55% absolute, +15–28% relative) and knee (males +31–44% absolute, +8–19% relative) than the ankle plantar flexors (males +20% absolute, no difference relative). In contrast the absolute differences between EM and EF were highly uniform across the five functional muscle groups investigated (absolute volume +47–53%). Relative to body mass, EM were also larger for total unilateral volume (+15%) and two muscle groups (HE +18%, KF +15%) than EF. By comparison there were no differences in relative muscle volume of the whole leg or functional muscle groups between EF and PMM_EF_. Furthermore, in support of our second hypothesis, relative hip extensor and knee flexor muscle volume, but not stature, percentage body fat or total relative muscle volume, differentiated the subgroups in accordance with their performance (i.e., EM > EF and PMM_EF_) rather than their sex (EM and PMM_EF_ vs. EF). This may indicate that greater hip extensor and knee flexor muscle volume of EM was not coincidental with sex, but was a likely cause of their faster sprint performance. Finally, it was observed that muscle volume distribution was more similar between elite groups from different sexes (EM and EF), rather than two groups of the same sex (EM and UM), suggesting that elite-level sprinters may share a common muscle distribution phenotype, that is independent of sex.

The full cohorts in the current study (males *n* = 31, females *n* = 22) were of a greater sample size than any previously published research comparing male and female sprinters (e.g., groups ≤9) (19, 20) while also being of a relatively high standard (SBE_100_ times of 111–112% of world record). The muscle volumes reported in the current investigation were in accordance with, although in many cases somewhat larger than, previously published data on male and female sprinters (15, 17, 19, 20).

### Male versus Female Differences

As anticipated, the cohort of male sprinters had 9% faster race performance than the female sprinters, but there were no differences in the relative performance standard of the cohorts (i.e., equivalent IAAF points and %WR). Similarly, as expected, the current study found male sprinters were taller, heavier, leaner, and had 34% greater fat-free mass than females, analogous to previous research (2, 3, 20, 22, 29). Indeed, similar sex differences were also observed between the elite sprint subgroups, with 9% faster race performance, and 45% greater fat-free mass of EM versus EF. It has been suggested that the performance difference between males and females could be a result of greater stature and associated limb lengths (21), lower relative fat mass, overall muscularity, or the muscularity of specific muscle groups (e.g., hip extensors). However, it has not been clear which of these factors is the key differentiator for the higher performance of males.

In this study, overall muscle volume was greater in the full male compared with full female cohort (absolute +38%, relative +14%), and similar differences were observed between the elite male and female subgroups (EM absolute +48%, relative +15%). More specifically, the full male cohort had greater muscle volume in 5 (absolute) and 4 (relative) functional muscle groups and 19 (absolute) and 12 (relative) out of 23 individual muscles/compartments compared with female sprinters. Correspondingly, EM had greater volume in 5 (absolute) and 2 (relative) functional muscle groups and 14 (absolute) and 2 (relative) individual muscles/compartments compared with EF. This sexual dimorphism with males having greater total muscularity than females is firmly established (10, 19, 20, 30–32) and is typically attributed to the higher levels of androgenic hormones, particularly testosterone, in males (33) and is considered the primary explanation for the greater neuromuscular force and power capability of males (1). Given that faster sprinting speeds are achieved by creating greater mass-specific ground forces during contact (34), as a consequence of muscular power production by the leg (35), the larger overall lower body muscularity observed in elite males would be expected to lead to a performance advantage for sprint running. Moreover, overall lower body muscularity in both absolute terms and relative to body mass has recently been highlighted as important for differentiating elite from subelite sprinters (11, 12). Therefore, it seems likely that the substantial between-sex differences in muscularity (both in full cohort and subgroup comparisons) observed in this investigation could largely explain the differences in sprint performance.

The sex difference in muscle volumes between the full cohorts was not uniform, but anatomically variable, with larger differences for the hip (male sprinters absolute +40–55%, relative +15–28%) and knee (male sprinters absolute +31–44%, relative +8–19%) muscle groups than the ankle plantarflexors (male sprinters +20% absolute, no difference relative). We have previously found that hip extensor and flexor muscularity to be particularly important for sprint performance (11, 12), and thus the observation of the most pronounced sex differences in muscularity occurring within these same muscles would appear to predispose male sprinters to faster performance.

### A Common Muscle Volume Distribution among Elite Sprinters, Irrespective of Sex

In contrast to the anatomical variability between the full male and female cohorts, for the elite groups the sex difference in volume of the functional muscle groups was almost uniform (absolute 47–53%, relative 15–20%), and consequently percentage of total muscle volume was also almost identical between the elite groups. This different pattern to the muscular differences among the full cohorts, but not elite sprinters, might suggest that elite sprinters are distinguished (i.e., attain elite sprint performance times) according to a common muscle distribution phenotype irrespective of sex. Furthermore, we also found the muscle volume distribution to be more similar between EM and EF (Fig. 5*C*) than between EM and UM (Fig. 5*D*), and as such, despite the sexual dimorphism in a wide array of human anatomy and physiology, elite male sprinters were shown to be more similar to a group of elite female sprinters than to another group of males (UM). Therefore, performance selection (i.e., being elite) appears to lead to greater similarity in muscle distribution than being of the same sex and further supports a muscle distribution phenotype specific to elite sprinting.

### What Accounts for the Sex Difference in Performance between Male and Female Sprinters?

The current investigation compared sprint performance, anthropometrics, and muscle volumes between three subgroups of elite males, elite females, and performance-matched (to elite females) subelite males. Figure 4 illustrates that sex was common between PMM_EF_ and EM, whereas absolute sprint performance was common between PMM_EF_ and EF. A potentially surprising finding [due to the widely observed sexual dimorphism where males have larger muscles than females (10, 31)] was the similarity of EF and PMM_EF_ for absolute and relative muscle volumes (no difference in total volume or any muscle group). Whereas EM had greater total absolute volume, ≥4 muscle groups, and ≥9 individual muscles/compartments when compared with PMM_EF_ and EF. Thus, the similar muscle volumes noted for EF and PMM_EF_ are in accordance with the similarity in sprint performance between these groups, and the greater muscle volumes observed for EM versus PMM_EF_ and EF reflect the faster sprint performance of this subgroup. Although both stature (3) and percentage body fat (22) have previously been suggested as being important for sprint performance (i.e., taller and leaner athletes are associated with faster performances), the current investigation observed that PMM_EF_ were in fact more similar to EM with regard to stature (0.06 m difference) and body fat percentage (3% different) than EF were to EM (stature, 0.14 m difference; body fat percentage, 10% difference). Consequently, these findings suggest that muscle volume, and specifically relative volume of the hip extensors, rather than stature or body fat percentage, may be the principal deterministic variable that explains the sex difference in sprint performance. However, it should be recognized that relative volume of the hip extensors is the absolute volume of the hip extensors divided by body mass, with the latter including overall muscle and fat mass. Therefore, the body composition candidate performance determinants we have considered are not discrete. Nonetheless, relative hip extensor muscle volume appears to best explain the performance of the three subgroups and thus the faster sprint performance of EM versus EF.

### Limitations

Although the comparison of male and female cohorts in this investigation used relatively large groups (males *n* = 31, females *n* = 22), the sample sizes for the subgroups were comparatively small (EM *n* = 5, EF *n* = 5, PMM_EF_ *n* = 6). By definition, elite-level athletes are rare and differ both quantitatively and qualitatively from subelite athletes as a result of being outliers in their given disciplines (36), and thus the group sizes for EM and EF are inherently limited and, in this case, represented a substantial proportion of elite-level sprinters in the United Kingdom at the time of the study. Furthermore, the control (i.e., unathletic) group used in this study was comprised only of male participants. A more comprehensive approach to comparing the differences in muscle volume distribution between untrained participants and elite sprinters would have been to also include untrained females for comparison with EF.

### Conclusion

In summary, for the full cohorts as expected male sprinters were more muscular than their female counterparts; however, the greater muscularity was nonuniform and anatomically variable, with the largest differences in the hip extensors and flexors that have been found to be the most important muscle groups for sprint performance. However, among elite sprinters the sex difference in volume of the functional muscle groups was almost uniform, with no differences in muscle distribution. This was coupled with the greater similarity of elite male sprinters to a group of elite female sprinters than to a group of untrained males and indicates for the first time that elite sprinters are selected for a common muscle distribution phenotype, irrespective of sex. The comparison of a group of male sprinters, performance matched to elite females, with elite males and females indicated that relative volume of the hip extensors, rather than other sex-based characteristics (e.g., stature and percent body fat), may be the primary determinant of the greater performance of elite males compared with elite females.

## DATA AVAILABILITY

Data will be made available upon reasonable request.

## GRANTS

This investigation was financially supported by UK Athletics and the UK Strength and Conditioning Association.

## DISCLOSURES

No conflicts of interest, financial or otherwise, are declared by the authors.

## AUTHOR CONTRIBUTIONS

R.M., M.J., S.J.A., and J.P.F. conceived and designed research; R.M. performed experiments; R.M., T.G.B., G.J.M., S.M., M.B.L., and B.H. analyzed data; R.M., M.J., S.J.A., and J.P.F. interpreted results of experiments; R.M. prepared figures; R.M. drafted manuscript; R.M., S.J.A., and J.P.F. edited and revised manuscript; R.M., T.G.B., S.M., M.B.L., B.H., M.J., S.J.A., and J.P.F. approved final version of manuscript.
